# Osteoporosis-related knowledge among students of a medical sciences university in Iran: calcium intake and physical activity

**Published:** 2015

**Authors:** M Ghaffari, M Nasirzadeh, S Rakhshanderou, M Hafezi Bakhtiari, J Harooni

**Affiliations:** * Environmental and Occupational Hazards Control Research Center, Iran; ** School of Public Health, Shahid Beheshti University of Medical Sciences, Tehran, Iran; *** Department of Public Health, School of Public Health, Rafsanjan University of Medical Sciences, Rafsanjan, Iran

**Keywords:** knowledge, Shahid Beheshti Medical Sciences University, osteoporosis

## Abstract

**Introduction:** Osteoporosis has increased the burden of diseases worldwide and in all populations. It is far more common among women than among men. The current research tried to investigate the health faculty students’ awareness of osteoporosis (calcium intake and physical activity).

**Materials & methods:** A descriptive-analytical research was conducted on 239 students in the health faculty, who voluntarily participated in this study and chose to use the census instancing approach. The information gathered employed a standardized questionnaire including 9 demographic questions, and 24 questions about osteoporosis were fed into SPSS 18 statistical software, t-test, Pearson’s correlation, and one-way ANOVA.

**Findings:** 228 students (95.4%) enrolled at the undergraduate level. They age averaged 22.17 ± 2.66. Their mean score of knowledge regarding osteoporosis was 12.96 ± 4.01 (4.67 ± 1.66 for calcium intake and 8.29 ± 2.89 for physical activity section). Only 46 (19.2%) students had a high-level knowledge of osteoporosis. There was a clear link (P < 0.05) among the students’ awareness regarding the marital status, being older, being a BA student of public health, and being a MA. There was no clear link among the awareness and the students’ job, parents’ job, education, and family income (P > 0.05).

**Discussion & conclusion:** Given the students’ field of study, that being health sciences, their awareness about osteoporosis was poor and unacceptable. Therefore, it was essential to promote the students’ knowledge and determine the causes of insufficient physical activity and calcium intake among students.

## Introduction

Osteoporosis is an illness in which the mass and intensity of bones are decreased and the risk of broken bones rises dramatically [**1**]. Osteoporosis is reported to be increasing in different parts of the world, affecting 75 million people in Japan, Europe, and the US [**2**]. The National Institute of Osteoporosis has estimated that more than 10 million people suffer from the disease; with further 33.6 million others who suffer from lower density of bones in their pelvis [**3**]. It is predicted that more than 50 per cent of bone breaking due to osteoporosis will be reported in Asia alone by 2050 [**4**]. The disease has not been diagnosed even in the majority of patients who have suffered breaking before, and as a result, it has gone untreated [**4**]. For example, in 2010, 50,000 cases of pelvis breaking were recorded in Iran, predicted to rise to 62,000 by 2020 [**4**]. Osteoporotic breakings are a cause of heavy economic load on families. In 2005, the Americans paid $ 17bn in prescription bills for osteoporotic bone breaks. With the aging population, it was predicted to nearly triple by 2040 [**3**]. 

The reliable data on the economic costs of the disease in Iran is missing; however, Soheili Azad et al. reported the costs of 16 days of hospitalization for a single break of the pelvis as being of $ 588 [**5**]. The disease affects all genders and races; its incidence rises with aging, being more prevalent in women than in men [**3**]. The lower bone density is a silent and progressive process, and when the bone breaks for the first time, it reveals no symptoms. Inactivity and neuromuscular disorders contribute to the progress of the disease [**1**,**6**]. Other factors such as age, female gender, family record, previous cases of break, being Caucasian, menopause, hysterectomy, long-term treatment with glucocorticoids (GCs), and rheumatoid arthritis are non-modifiable risk parameters. Also, smoking and alcohol consumption, low body mass index, poor diet and inadequate intake of calcium and vitamin D, and poor awareness on the issue, eating disorders, lack of exercise, and frequent falls are modifiable risk factors directly affecting bone tissue and contributing to the minerals mass [**7**,**8**]. Public awareness of osteoporosis is poor especially in the underdeveloped countries. Health education programs are reported have an influence on improving the general knowledge and sustaining it in the long run [**9**]. While working on female students to find their awareness of osteoporosis in Kolaleh, Gorgan, a northern city of Iran, in 2012, Ghaffari et al. reported that 55 per cent of the students had a poor awareness of the disease [**10**]. In another study on female students in middle school in Andisheh Township, a Tehran’s suburb, they found that the general awareness was extremely poor [**11**]. Bearing this in mind, the current research tried to evaluate the osteoporosis knowledge of students in health faculty, Shahid Beheshti University of Medical Sciences, to provide the necessary interventions in health education programs.

## Materials & Methods

A research of the female students in the health faculty, Shahid Beheshti University of Medical Sciences, in the 2011-12 educational year was conducted with a consensus sampling method, and 239 questionnaires out of all the 400 questionnaires distributed randomly among them, were filled in. A valid and reliable questionnaire with two sections, used by Baheiraei et al. [**12**] was the data collection tool. The first section included 9 demographic information questions and the second one had 24 questions regarding the students’ awareness of osteoporosis (including 9 in calcium intake and 15 others in physical activity). The Cronbach’s alpha coefficient on calcium intake and physical activity was reported as 0.55 and 0.66, respectively [**10**]. 

The correct answers were assigned the score 1 and incorrect answers and answers of “don’t know” were assigned the point of zero. The total score of calcium intake fell into a range of 0-9, and that of physical activity fell into a range of 0-15, thus with the total score falling into an overall range of 0-24. Drawing upon Ghaffari et al., and based on scores, the students’ awareness was assigned into three categories of high (16-25), average (8-16), and poor (0-8). The researchers provided the students with the objectives and scope of the study and asked them to take part in the study on consent. The data collected were fed into SPSS 18 statistical software and investigated by using the Pearson’s relation of factor, independent t-test, and one-way variance investigation. 

## Results

**Fig. 1:**
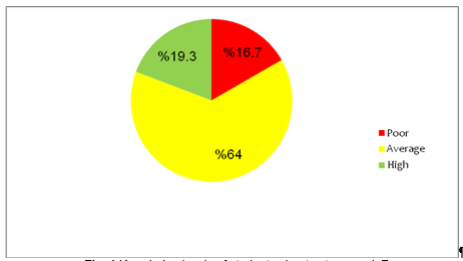
Knowledge levels of students about osteoporosis

Of 239 participant students, 228 (95.4 per cent) enrolled in BA. They averaged between 22.17 ± 2.66 regarding age (18-24 years old). 219 students (91.6 per cent) were married and 20 (8.54 per cent) were single. 66 (27.6%) of the male parents and 31 (13%) of the female parents had a university degree. 210 female parents (87.9 per cent) were housewives and 136 male parents (56.9 per cent) had white-collar jobs. Only 5.4 per cent of the students reported their income level as quite high (**Table 1**). The students’ mean score of osteoporosis awareness was 12.96 ± 4.01 (4.67 ± 1.66 for calcium intake and 8.29 ± 2.89 for osteoporosis awareness). **Tables 2** and **3** show the frequency distribution of the students’ responses to questions regarding their awareness of calcium intake and physical activity. Only 46 students (19.2) scored high in their awareness (**Fig. 1**). A clear relation was realized among osteoporosis and the other variables including the marital status, being older, being a student of public health, and the degree to be awarded (P < 0.05). No significant correlation was found between the students’ jobs, parents’ jobs, education, and the income level (P > 0.05) (**Table 4**).

**Table 1:** Students’ demographic information

Variable			Demographic distribution		
University major	Number (per cent) Public Health	Number (per cent) Occupational Health	Number (per cent) Environmental Health	Number (per cent) Health Education	Number (per cent) Epidemiology
	(58) 3/24	(51) 3/21	(121) 6/50	(5) 1/2	(4) 7/1
Degree to be awarded	BA		MA		PhD
	(228) 4/95		(9) 8/3		(2) 8/0
Father’s education	No education	Grade school	Middle school	High school diploma	University degree
	(18) 5/7	(38) 9/15	(44) 4/18	(73) 5/30	(66) 6/27
Mother’s education	No education	Grade school	Middle school	High school diploma	University degree
	(21) 8/8	(60) 1/25	(49) 5/20	(78) 6/32	(31) 13
Father’s job	Unemployed	Blue-collar job	White-collar job	Other	
	(8) 3/3	(13) 4/5	(136) 9/56	(82) 4/34	
Mother’s job	Housewife		White-collar job		Other
	(210) 9/87		(6) 5/2		(23) 6/9
Family income*	Low	Average	Good	High	
	(12) 5	(61) 5/25	(153) 64	(13) 4/5	
Marital status	Single				Married
	(219) 6/91				(20) 4/8
Students’ job	Employed				Unemployed
	(17) 1/7				(222) 9/92
* low = insufficient for living; average = below sufficient level; good = sufficient for living; high = well above the sufficient level.					

**Table 2:** The distribution of students’ responses to osteoporosis questions: calcium intake

Questions	Correct response		Incorrect response		Don't know	
	Number	Percent	Number	Percent	Number	Percent
1. Consumption of less dairy products increases the likelihood of osteoporosis.	215	90	20	3/8	4	7/ 1
2. Appearance of menopause increases the likelihood of osteoporosis.	190	5/79	7	9/2	42	9/17
3. A greater body size decreases the likelihood of osteoporosis.	49	5/20	73	5/30	117	49
4. A higher consumption of broadleaf vegetable (spinach) decreases the likelihood of osteoporosis.	12	5	164	6/68	63	4/26
5. Having a mother or a grandmother with osteoporosis increases the likelihood of the disease.	78	6/32	76	8/31	85	6/35
6. Blonde and bright skin increases the likelihood of osteoporosis.	57	8/23	54	6/22	128	6/53
7. Hysterectomy operation increases the likelihood of osteoporosis.	101	3/42	20	3/8	122	51
8. Long-term intake of cortisol increases the likelihood of osteoporosis.	96	2/40	21	8/8	122	51
9. Regular physical exercise decreases the likelihood of osteoporosis.	7	9/2	216	4/90	16	7/ 6

**Table 3:** The distribution of the students’ responses to osteoporosis regarding physical activity

Questions	Correct response		Incorrect response		Don't know	
	Number	Percent	Number	Percent	Number	Percent
1. High-speed walking reduces osteoporosis incidence.	63	4/26	92	5/38	84	1/35
2. Cycling reduces osteoporosis incidence.	117	49	30	5/12	92	5/38
3. To improve bones, at least 3 sessions of physical exercise are necessary.	6	5/2	191	9/79	42	6/17
4. The required time for the exercise is of 45 minutes and more.	15	3/6	170	1/71	54	6/22
5. To improve bones, physical exercise with an average breathing speed is enough.	105	9/43	65	2/27	69	9/28
6. Slow running is a good measure to decrease osteoporosis indigence.	157	7/65	13	4/5	69	9/28
7. Aerobic exercise is a good measure to decrease osteoporosis incidence.	7	9/2	161	4/67	74	7/29
8. Cheese is a good calcium source.	5	1/2	216	4/90	18	5/7
9. Sardines are a proper source of calcium.	3	3/1	168	2/70	68	5/28
10. Cabbage (broccoli) is a proper calcium source.	31	13	150	7/62	58	3/24
11. Yogurt is a proper calcium source.	196	82	30	5/12	13	4/5
12. Ice-cream is a proper calcium source.	162	8/67	41	1/17	36	1/15
13. Adolescents should have an intake of 1200-1300 mg of calcium per day.	12	5	101	2/42	127	7/52
14. Two glasses or more of milk are enough to provide body with calcium.	7	9/2	215	9/89	17	1/7
15. Calcium supplements should be taken if calcium is insufficient in the body.	27	3/11	168	2/70	44	4/18

**Table 4:** Mean and standard deviation of students’ awareness in terms of variables

Variable			Mean and standard deviation			P. Value
University major	Public Health	Occupational health	Environmental Health	Health Education	Epidemiology
	5/ 3± 3 /15	01/ 4± 9 /10	3 /6± 5 /12	6/ 1 ±4/ 15	3 /5 ±2/ 15	P<0.001
Degree to be awarded	BA		MA		PhD	
	9 /3 ±8/ 12		7 /1± 2/ 16		1/ 9± 5/ 14	P=0.03
Father’s education	No education	Order school	Mid school	High school diploma	University	
	5/ 3 ±5 /12	8/ 3± 7/ 13	9/ 3± 1/ 13	8/ 3 ±02 /13	5 /4± 4/ 12	P=0.76
Mother’s education	No education	Grade school	Middle school	High school diploma	University degree	
	6/ 3± 4/ 13	8/ 3± 7/ 12	04/ 4 ±6/ 13	19/ 4 ±01/ 13	01/ 4 ±8/ 11	P=0.35
Father’s job	Unemployed	Blue-collar job	White-collar job	Other		
	7/ 3 ±3/ 12	3/ 2± 2 /11	9 /3 ±7/ 12	2/ 4 ±6/ 13		P=0.12
Mother’s job	Housewife		White-collar job		Other
	9/ 3± 9/ 12		4/ 3± 8 /12		5 /4± 8/ 12	P=0.33
Family income	Low	Average	Good	High		
	8/ 3 ±6 /11	9/ 3 ±3/ 13	08 /4± 9/ 12	6 /3 ±6 /12		P=0.58
Marital status	Single				Married	
	04 /4 ±7 /12				2 /3 ±8 /14	P=0.01
Students’ job	Employed				Unemployed	
	4 /4± 1/ 14				9/ 3± 9 /12	P=0.27

## Discussion

Osteoporosis has increased the burden of diseases worldwide and in all populations [**13**]. It is far more common among women than among men [**13**]. It is less known as a disease than as a risk factor to bone breaking including breaks in femur, pelvis, and ribs [**13**]. The current research was an investigation performed to evaluate the health faculty students’ awareness of osteoporosis (calcium intake and physical activity). 

A total score of 12.96 out of 24 was found, with an average-level score of 4.67 out of 9 for the awareness of calcium intake and 8.29 out of 15 for physical activity. Only 19.2 per cent of the participants scored high in their awareness of osteoporosis, with an average score for more than half of participants. Kimberley et al. (2009) reported similar scores of 14.61 out of 24 for the awareness of osteoporosis in a group of students [**14**]. Other studies reported a poor awareness of young female students [**10**,**15**,**16**]. The examination of the individual questionnaire items found a high awareness of students regarding the effect of a lower consumption of dairy products in daily diet and menopause on the incidence of osteoporosis; but it did find a low awareness regarding the effect of consumption of broadleaf vegetables and knowledge of calcium sources. These findings were similar to those of Hazavei et al. (2004) and Ghaffari et al. (2013) [**10**,**17**]. Given the students’ poor knowledge of osteoporosis, interventions based on the students’ responses to the questionnaire in order to improve their awareness, seems necessary. The findings in this research and in other similar studies recommended policies leading to the prevention of osteoporosis and thus frequent bone breakings in elderly people. Ghaffari et al. reported a high level of awareness about yogurt as a good source of calcium for 58 per cent of students, but a poor awareness about other sources of calcium, similar to our findings [**10**]. Similar to our study, Charlot and Kathy (2007) reported most of the responses for items related to the ways of preventing osteoporosis; but they reported a poor score in the identification of risk factors [**18**]. Ghaffari et al. [**10**] also reported that 8.6 per cent of the participants were unaware of the fact that having a small body size is regarded as a potential risk factor of osteoporosis; we reported that only 2.5% of the participants were unaware of the fact. However, Jean and Cynthia reported that 56% of the students were aware of this fact [**15**]. A large number of researches in Iranian context have focused on students; therefore, it is recommended that more research examined the osteoporosis awareness and the determinant factors in university students.

We also found that 25% of the members had a good stage of awareness about the different physical exercises to prevent osteoporosis. Larkey et al. [**17**] and Hazavei et al. [**19**] similarly reported a poor level of awareness among women aged 25-55 years and students, about the different types of physical exercises that could help prevent osteoporosis. Our findings also indicated that few numbers of students (23.9 per cent) perceived the white skin as increasing the probability of osteoporosis. Ghaffari et al. [**10**] and Mirza Aghaei et al. [**20**] reported that participants did not respond to the question accurately. We reported a very low score for the participants’ awareness of rich sources of calcium. Similarly, Ungan and Tumer [**21**] found that only 36 per cent of women identified sources rich in calcium. In our study, only 5 per cent of the students were familiar with the daily body need for calcium of 1200-1300 mg/ day, which was evaluated as poor, given the fact that the participants came from a medical sciences background. The study recommended training workshops about the risk factors and prevention measures of osteoporosis in health faculty. 

A significant correlation was reported between awareness and being older, married, student of public health, and the degree to be awarded as MA; however, this correlation was not significant between the awareness and the students’ jobs, parents’ education and job, and family income, which was in line with findings in Ghaffari et al. [**10**]. It is likely that the students’ awareness of osteoporosis is associated with individual factors such as older ages and university major. Thus, we recommend further research in order to examine osteoporosis awareness and its different determinants (individual, social, and familial). 

## Conclusion 

Our findings indicated a relatively unsatisfactory level of awareness among participants regarding osteoporosis (calcium intake and physical activities), with a further impact on their unsatisfactory attitudes and performance in adopting behaviors to prevent osteoporosis. Given that our participants were students of health sciences, who were directly related to the research area, we evaluated their awareness of the issue as unacceptable and poor. We recommend more research on other areas such as other determinant factors of osteoporosis awareness, in addition to proper interventions, so the necessary policy actions and educational intervention could be made at the national level. 

**Acknowledgement**

The authors would like to thank the staff, students, and professors of the faculty of health, Shahid Beheshti University of Medical Sciences for their help and participation in the research.
